# Impact of Teacher Incentive Intervention on Students’ Vision Healthcare Uptake: A Cluster-Randomized Controlled Trial

**DOI:** 10.3390/ijerph191912727

**Published:** 2022-10-05

**Authors:** Jin Zhao, Huan Wang, Hongyu Guan, Kang Du, Yunyun Zhang, Nathan Congdon

**Affiliations:** 1 School of Marxism, Xi’an University of Finance and Economics, Xi’an 710110, China; 2 Freeman Spogli Institute for International Studies, Stanford University, Stanford, CA 94305, USA; 3 Center for Experimental Economics for Education, Shaanxi Normal University, Xi’an 710110, China; 4 School of Economics, Xi’an University of Finance and Economics, Xi’an 710110, China; 5 Centre for Public Health, Queen’s University Belfast, Belfast BT7 1NN, UK; 6 Zhongshan Ophthalmic Center, Sun Yat-sen University, Guangzhou 510000, China

**Keywords:** cluster-randomized controlled trial, rural China, school-aged children, uptake, vision care

## Abstract

Less than one-third of rural Chinese children with refractive error own or wear eyeglasses. To study the effect of teacher incentives on the acceptance of vision care offered to rural students with uncorrected refractive error, we conducted a cluster-randomized controlled trial in 18 townships in one county in Shaanxi Province. Primary and junior high schools within each township were assigned to either intervention (all teachers received an incentive) or control (no teacher incentives were offered) groups. A total of 42 schools were assigned to either the intervention group (13 schools) or the control group (29 schools). Teachers in the intervention group could elect to receive high-value (sunglasses worth USD 148), moderate-value (eyeglasses worth USD 89), or cash incentives (USD 35) if ≥70% of eligible students (uncorrected visual acuity (VA) ≤ 6/12 in both eyes and corrected VA ≤ 6/9.5 in both eyes) in the teacher’s class visited a program-affiliated vision center (VC) within 60 days after their vision screening. Among 8238 students, 3401 (41.2%, of which 53.0% were girls with a mean age of 12 (SD 1.75)) met the enrollment criteria and were randomly allocated to the intervention (n = 1645, 49.0%) and control groups (n = 1579, 51.0%). Among these, 3224 (94.8%) completed the study and underwent analysis. Nearly equal numbers of students had classroom teachers selecting the high-value (n = 524, 31.9%), moderate-value (n = 582, 35.4%), and cash incentives (n = 539, 32.8%). The rate of the acceptance of offered vision care was significantly higher in the intervention group (382/1645 = 23.2%) compared to the control group (172/1579 = 10.9%, 95% confidence interval for observed difference 12.3%, *p* < 0.001). Teacher incentives appeared effective in improving Chinese rural school-aged children’s uptake rate of vision services provided by county hospital-based VCs.

## 1. Introduction

A series of World Health Organization (WHO)-supported studies have suggested that approximately 10–20% of school-aged children in developing countries suffer from vision problems—principally refractive error—that may be safely and inexpensively corrected by eyeglasses [1,2,3,4]. Nearly half of the world’s school-aged children with unresolved refractive error live in China [5]. Despite the practicality of vision screening and the ease of refractive error correction with eyeglasses [1,6], in areas such as rural China, less than one-third of the children who need them own or wear eyeglasses [1,3,4,7,8]. 

Beyond enhancing children’s quality of life by improving their vision, providing children with eyeglasses has been shown to significantly improve their educational performance [6,7]. In fact, the effect of eyeglasses on children’s academic outcomes has been shown to be at least as large as that of educational interventions widely deemed to be successful [9].

There exist many factors behind the low rate of eyeglasses ownership and usage in underserved areas such as rural China. Although the cost of eyeglasses has been identified as a factor keeping the lowest-income families from affording eyeglasses, many parents are willing and able to purchase eyeglasses for their children [10,11,12,13,14,15]. However, prior research has suggested that misinformation about the safety of eyeglasses for children’s vision and a lack of awareness about the importance of wearing eyeglasses for vision health contribute more to low eyeglasses ownership and usage rates than cost burdens [14]. Another significant factor is the lack of access to high-quality vision healthcare services in rural China [16]. For example, rural children and their families may be unaware of the process for obtaining their first pair of eyeglasses. As a result, eyeglasses ownership remains limited in the rural areas of China [13,17]. In 2010, an estimated 215 million rural residents were found to be visually impaired for this reason [13,18,19].

Underscoring the importance of providing refractive care for China’s population, President Xi Jinping recently announced plans for a comprehensive national children’s myopia management project. Actions will be coordinated among eight central government bodies under the leadership of the Ministry of Education [20]. However, for China’s central government to effectively implement such a large-scale project [21], it is necessary to research viable ways of providing low-income families with high-quality refractive services and access to eyeglasses.

One of the most popular strategies used by non-governmental organizations and governments for overcoming barriers to accessing high-quality eyeglasses is the community-based vision center (VC) [18,22]. VCs are fixed facilities that provide affordable and sustainable eyeglasses and eye care services for local communities, and generally cater to people with uncorrected refractive error [22]. In addition, recent evidence suggests that VCs can positively affect children’s academic performance and improve their rates of eyeglasses ownership and wear [4,21,23].

Despite the popularity of VCs, they have demonstrated low rates of vision service uptake among children with uncorrected refractive error. We believe that the general lack of knowledge about vision problems and ways to correct them at the family level are important contributing factors [14,24]. Moreover, results from recent studies have suggested that teacher-based interventions may be helpful for addressing these issues and for improving the rates of vision service uptake for school-aged children [25,26,27]. Not only has it been shown to be easier and less expensive for teachers to administer treatment to their students, but as most, if not all, school-aged children in the community attend school, the information conveyed by a teacher to a student’s parent is also more likely to attract parental attention and motivate parents to act [24,28]. Additionally, incentives directed at teachers have been shown in previous clinical trials to improve children’s uptake of eyeglasses [27]. Teachers in rural China can also accurately perform vision screening for students with only moderate training [29,30].

In the current study, and based on previous studies on children’s vision problems [4,6], we hypothesize that providing teachers with incentives for engaging with the parents of children tested to have refractive error will help to improve students’ acceptance of offered eye examinations and services at VCs (i.e., this study’s main outcome), as well as improve the rate of eyeglasses uptake in VCs. Therefore, this study attempts to fill in the gap by providing quantitative results on the impact of providing teacher incentives for improving rural students’ visual healthcare acceptance.

## 2. Materials and Methods

The protocol for this study was approved in full by the Institutional Review Boards at Stanford University (Palo Alto, CA, USA; Reference Number 52514) and the Zhongshan Ophthalmic Center (ZOC; in Guangzhou, China). Permission was received from the local Boards of Education and the principals of all involved schools. The principles of the Declaration of Helsinki were followed.

### 2.1. Vision Center Set-Up and Staff Training 

We established a VC in the county-level hospital of a nationally designated poverty county located in the middle of Shaanxi Province; this hospital was the only public medical institution providing refractive services in the area. As nationally designated poverty counties are recipients of a variety of government-sponsored poverty alleviation programs, the county selected for our study provides an effective representation of poor counties in rural China. In 2015, Shaanxi’s provincial per capita gross domestic product (GDP) was RMB 48,135 (or USD 7728), and the total population of the sample county was 188,500 with a per capita GDP of RMB 26,752 (or USD 3889) [31]. Additionally, the sample county ranked 73rd out of 107 counties in Shaanxi Province in 2015, with a per capita GDP lower than the typical county in Shaanxi (RMB 48,836, or USD 6927) [32].

The VC was established in collaboration with the provincial and prefectural Bureaus of Education. The county hospital selected three employees (one ophthalmologist and two ophthalmic nurses) to become staff in the VC. These individuals underwent one month of formal refraction training (from September to October 2014) at Zhongshan Ophthalmic Center in Guangzhou, China. At the end of the program, all three employees were certified as qualified optometrists and dispensing opticians by China’s Ministry of Labor and Social Security. After this formal training, the county staff members underwent one month of supervised practical training in the VC; during this time period, each staff member screened and measured for refractive error in hundreds of children from local schools and received practical instruction for distributing eyeglasses. A consultant from the Brien Holden Vision Institute provided management training, including inventory control and record keeping.

### 2.2. Data Collection

At baseline, the research team surveyed 42 primary and junior high schools in the sample county. All students in grades 4 through 6 in 29 primary schools and grades 7 through 9 in 13 junior high schools were included. In November 2016, to begin program implementation in each school, teachers administered a socioeconomic survey to all students. The socioeconomic survey collected data on students’ age, sex, eyeglasses ownership, the education level and migration status of the parents, and the distance between the town and county seats. Baseline eyeglasses ownership was defined as a student bringing a pair of eyeglasses to school after being asked to bring them. In addition, all program teachers were surveyed on their teaching experience, their attitude towards wearing eyeglasses, and their vision care knowledge.

Our sample provides efficient statistical power to detect the impact of our trial. Power calculations were conducted using Optimal Design software for cluster randomization. We determined that 16 towns with 515 students per town (with 155 expected to have refractive error) conferred 80% power, with an α of 0.05, intraclass correlation of 0.15, and explained variation by covariates (R2) of 0.50, to detect a difference of 0.15% in follow-up acceptance of offered vision care between the study’s intervention arms and the control group.

### 2.3. School-Based Visual Acuity Assessment and Eligibility Criteria 

Visual acuity (VA) screening was tested separately for each eye without refraction at 4 m using Early Treatment Diabetic Retinopathy Study (ETDRS) eye charts (Precision Vision) in a well-lit room [8,31]. Children who owned eyeglasses were requested to bring them to school, and during the screening, their VA was tested both with and without their eyeglasses. If the orientation of at least 4 of 5 optotypes on the 6/60 line was correctly identified, children were examined on the 6/30 line, on the 6/15 line, and then line by line to 6/3. VA for one eye was defined as the lowest line on which 4 of 5 optotypes were read correctly. If the topmost line could not be read at 4 m, the participant was tested at 1 m, and the measured VA was divided by 4.

In this study, we define normal vision as uncorrected (no eyeglasses) VA > 6/12 in both eyes or corrected (wearing eyeglasses) VA > 6/9.5 in both eyes. Failing the visual screening was defined as uncorrected VA ≤ 6/12 in either eye or corrected VA ≤ 6/9.5 in either eye. All children who failed the VA screening were referred to the county hospital VC for further examinations and were enrolled into the trial.

To calculate and compare different visual acuity levels, we require a linear, continuous scale with constant increments [4]. In the field of ophthalmology/optometry, LogMAR is one of the most commonly used continuous scale. This scale uses the logarithm transformation: LogMAR = log10(MAR). In this definition, the variable MAR (Minimum Angle of Resolution) is defined as the inverse of VA: MAR = 1/VA. LogMAR offers a relatively intuitive interpretation of VA measurements. It has a constant increment of 0.1 across its scale, and each increment indicates approximately one line of VA loss in the ETDRS chart. Thus, the higher the LogMAR value, the worse one’s vision [4]. 

### 2.4. Acceptance of Offered Vision and Eyeglasses Uptake

Our analysis focused on one key variable: acceptance of offered vision care. The follow-up survey was conducted within sixty days after the VA screening at each school.

The primary outcome was defined as the students who failed the vision screening (and thus were detected to have uncorrected refractive error) and went on to accept the vision care offered to them by the time the follow-up survey was conducted. We defined acceptance of offered vision care as a binary variable taking the value of 1 if parents brought their child to the VC for services during the duration of the trial.

Our analysis also concerned eyeglasses uptake. The secondary outcome was defined as the acceptance of offered eyeglasses if a student needed to wear eyeglasses for vision correction after their vision screening in the VC. We defined eyeglasses uptake as a binary variable taking the value of 1 if the student accepted the offered eyeglasses during the duration of the program.

### 2.5. Treatment and Experiment Design

The parents of the students who failed the vision screening received a letter that described the VC program and invited them to bring their children to the VC for a free examination that included re-screening and refraction correction. Eyeglasses were provided to primary school students for free and were available for purchase at a subsidized price (USD 30) for junior high school students. Eyeglasses were provided for free for primary school students for two reasons: to get young children used to wearing eyeglasses, and to overcome the existing stigma against eyeglasses wear that is prevalent among children in this age group. Teachers assisted the VC staff throughout the VA screening and referral process. Teachers also received a list of students who failed the vision screening. Finally, the VC staff made follow-up phone calls to teachers and families to encourage parents to bring their children to the VC for services.

The study was conducted as a cluster-randomized clinical trial, with randomization occurring at the township level. Schools were paired based on similar distances from the county seat and on the number of students enrolled. Schools in these pairs were randomly assigned to one of two groups: the intervention group (that would receive teacher incentives) or the control group (that would receive no teacher incentives). In the intervention group, a member of the research team signed a “teacher incentives agreement” with the teachers. Teachers in the intervention group chose one of three types of incentives listed in the agreement: high-value incentives (a pair of sunglasses worth USD 148), moderate-value incentives (a pair of eyeglasses worth USD 89), and cash incentives (USD 35 in cash). Teachers in the intervention group would receive the incentive they chose if more than 70% of their students with uncorrected refractive error visited the VC within sixty days after their vision screening. In the control group, teachers were not offered any incentives. Teachers in the control group also did not know about the system of incentives. 

**Figure 1:**
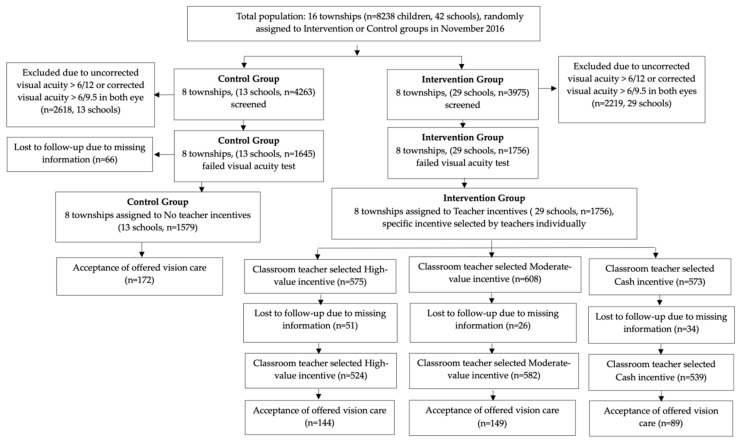
Schematic diagram describing the sample for the study.

All participants (teachers, parents, and students), as well as the VC staff, were not informed of the study design or of group assignment. Participants were told that the study was on vision care among children; accordingly, teachers were unaware of their participation in the trial and were masked to group assignment during the follow-up outcome assessment. Therefore, although teachers in the intervention group signed an incentive agreement and chose the incentive they wished to receive, they were not aware that they were participating in a trial; on the other hand, teachers in the control group were not aware that incentives were being offered as part of the program (for the intervention group). Figure 1 shows the research design of this study.

### 2.6. Tests for Balance and Attrition Bias

Among the 8238 children at 42 randomly chosen schools in 16 townships, 4837 (58.7%) were excluded based on having uncorrected VA higher than 6/12 (VA > 6/12) and corrected VA higher than 6/9.5 (VA > 6/9.5) in both eyes, and 3401(41.3%) were found to require eyeglasses. There were 1765 (51.9%) students in 29 sample schools who were randomly assigned to the intervention group and 1645 (48.1%) students in 13 sample schools who were randomly assigned to the control group.

Among the 3401 children enrolled and assigned to study groups, 177 (5.2%) were lost to follow-up due to missing data at baseline. This left a final analytic sample of 3224 students: 1645 (48.4%) students in the intervention group and 1579 (51.6%) in the control group. There was no significant difference between the two student groups in regard to their sex, their eyeglasses ownership status at baseline, the severity of their myopia (measured by the LogMAR of the worse eye), their parents’ education status (defined as at least one parent with nine or more years of education), their parents’ migration status (defined as at least one parent out-migrating for work), if they attended primary school (versus junior high), the distance between their town and county seat, the teaching experience of their teacher, their teacher’s support of myopic students wearing eyeglasses in the classroom, their teacher’s belief in eye exercises (i.e., whether their teacher believed eye exercises can treat myopia or not), their teacher’s belief in the impact of myopia (i.e., whether their teacher believed that not wearing eyeglasses impacted a myopic student’s achievement or not), and their teacher’s beliefs for requiring eyeglasses (i.e., whether their teacher believed that low degrees of myopia required eyeglasses for treatment or not).

### 2.7. Statistical Analysis

We first conducted a general descriptive analysis of the impact of different incentives on student acceptance of offered vision care. We then used a one-way variance regression model to explore potential factors determining students’ acceptance of offered vision care. Third, we used a multiple logistic regression model to measure the odds ratio of teacher incentives on a student’s acceptance of offered vision care at follow-up, adjusting for other baseline student characteristics, including student’s age, sex, eyeglasses ownership status at baseline, VA of the worse eye at baseline, if they had at least one parent with nine or more years of education, if they had at least one parent out-migrating for work, if they attended primary school (versus junior high), the distance between their town and county seat, their teacher’s teaching experience, if their teacher supported myopic students wearing eyeglasses, and their teacher’s vision knowledge.

All analyses were performed using STATA version 14.2. We calculated robust SEs to adjust for clustering by township. Considering potential characteristics that were not fully balanced by randomization, we prespecified that all tests were two-sided tests, and a *p*-value less than 0.05 was considered statistically significant. These characteristics were a student’s age, the severity of their myopia (as measured by the LogMAR of the worse eye), their parents’ migration status (defined as at least one parent out-migrating for work), if they attended primary school (versus junior high), and the distance between their town and the county seat.

To reduce the inefficiency of estimation due to missing values, we used multiple imputations in STATA to impute missing data for two characteristics at baseline: at least one parent with nine or more years of education (n = 127), and at least one parent out-migrated for work (n = 117). We used logistic regression for binary and ordinal variables. The independent variables used for imputation included all non-missing variables listed in Table 1. The multiple imputation approach created 67 copies of the data in which missing values were imputed by averaging these 67 datasets using Rubin’s rules, which ensured that the standard error for all regression coefficients accounted for the uncertainty in the imputations and estimation.

## 3. Results

This section reports the four main findings of our analysis. Table 2 presents the descriptive statistical analysis of the impact of teacher incentives on student acceptance of offered vision care and the eyeglasses uptake rate. Table 3 presents the primary analysis, in which we used univariate analysis to examine the association between teacher incentives and student acceptance of offered vision care, and then used multiple logistic regression to determine whether students in the intervention group had a higher acceptance of offered vision care compared to students in the control group. Table 4 presents the logistic regression analysis of the impact of teacher incentives on the acceptance of offered vision care between primary and junior high school groups. Table 5 presents the logistic regression analysis of the impact of the various incentives on student acceptance of offered vision care.

First, Table 2 presents students’ acceptance of offered vision care in the intervention and control groups, as well as the acceptance rates of the intervention subgroups and the eyeglasses uptake rate in the different groups. The descriptive analysis showed that the average student acceptance of offered vision care in the intervention group was 12% (*p* < 0.001) higher than that of the control group (Table 2). Within the intervention group, the average student acceptance of offered vision care in the high- and moderate-value incentives subgroups was 17% (*p* < 0.001) higher than that of the control group. There were no significant differences between the cash incentives subgroup and the control group in student acceptance of offered vision care.

In addition, when eyeglasses uptake was stratified by the study groups, 77% (268/347) of students accepted eyeglasses and offered vision care in the intervention group and 86% (135/161) of students accepted eyeglasses and offered vision care in the control group. Of the students who accepted offered vision care in the intervention subgroups, the average eyeglasses uptake of the high- (105/131) and moderate-value (110/133) incentives subgroups was 81% and 83%, respectively. In the cash incentives subgroup, 64% (53/83) accepted eyeglasses. Finally, there were significant differences between the cash incentives subgroup and the control group in regard to students’ acceptance of eyeglasses.

In the multivariate regression model, teacher incentives were highly associated with a student’s acceptance of offered vision care (OR = 1.94, [CI] 1.57–2.38, *p* < 0.001) (Table 3). Other variables significantly associated with a student’s acceptance of offered vision care included age (OR = 0.89, [CI] 0.81–0.99, *p* = 0.028), being of the female sex (OR = 1.36, [CI] 1.12–1.65, *p* = 0.002), having a VA in the worse eye at baseline (OR = 1.95, [CI] 1.25–3.04, *p* = 0.003), having at least one parent out-migrating for work (OR = 0.81, [CI] 0.67–0.99, *p* = 0.038), being a primary school student (OR = 2.04, [CI] 1.40–2.98, *p* < 0.001), the distance between their town and county seat (OR = 1.01, [CI] 1.00–1.02, *p* = 0.016), and if their teacher believes that myopic students not wearing eyeglasses worsens school performance (OR = 1.49, [CI] 1.02–2.18, *p* = 0.037). Finally, factors such as eyeglasses owned at baseline, their parents’ education status, their teacher’s teaching experience, if their teacher supports myopia students wearing eyeglasses, if their teacher believes that eye exercises cannot prevent myopia, and if their teacher believes that students with low degrees of myopia should wear eyeglasses were not associated with a student’s acceptance of offered vision care.

In addition, although excluding those characteristics that are imbalanced in the baseline survey, the impact of providing teacher incentives on students’ acceptance of offered vision care is still significant (OR = 2.46, [CI] 2.02–2.99, *p* < 0.001) (Table 3).

In the multivariate regression model, teacher incentives also had an impact on the acceptance of offered vision care in students with uncorrected refractive error among the primary schools group (OR = 2.18, [CI] 1.64–2.90, *p* < 0.001) and the junior high schools group (OR = 1.89, [CI] 1.35–2.66, *p* < 0.001) (Table 4).

We also found that there was a difference in impacts among the different incentive subgroups on the acceptance of offered vision care among students with uncorrected refractive error (Table 5). (We note that though the incentive subgroups were roughly equal in size, this was due to chance alone; teachers were free to select among any of the three offered incentives.) Specifically, as shown by the multivariate regression model, high-value incentives (OR = 1.67, [CI] 1.32–2.11, *p* < 0.001) and moderate-value incentives (OR = 1.78, [CI] 1.42–2.23, *p* < 0.001) significantly improved students’ acceptance of offered vision care. However, providing cash incentives did not affect students’ acceptance of offered vision care.

## 4. Discussion

In this study, we documented the high prevalence of uncorrected refractive error in school-aged children using the results from a visual acuity screening among 8238 students in grades 4 through 6 and 7 through 9 in 42 primary and junior high schools in poor, rural parts of Shaanxi Province. At baseline, 41.3% of children in the sample had uncorrected refractive error. This finding is consistent with the rate reported by previous studies inside and outside of China [2,7,8,15].

Given the high prevalence of uncorrected refractive error reported in our results, the rates of eyeglasses use we observed are quite low. Only 37.3% of our sample brought eyeglasses to school. These findings raise several important concerns regarding vision care in rural areas. It is possible that children (or their parents) either do not know that they have a vision problem or do not know how to address their vision problems [33], which, combined with the common misconception among rural communities that eyeglasses have negative impacts on vision, suggests that poor vision knowledge and misinformation may be the cause of the limited ownership and use of eyeglasses observed in this study.

We did, however, detect a significant improvement in students’ (with uncorrected refractive errors) acceptance of offered vision care in the intervention group. The acceptance rates of offered vision care for the intervention and control groups were 23.2% and 10.9%, respectively. Additionally, teacher incentives had an impact on a student’s acceptance of offered vision care, as shown by our multivariate regression model. Furthermore, the average eyeglasses uptake of students who accepted the offered vision care in both the intervention group and the control group was 82%. Additionally, teacher incentives improved both primary and junior high school students’ acceptance of offered vision care, even though eyeglasses were not provided for free for junior high school students. This result suggests that teacher incentives are effective at increasing healthcare uptake in settings where care is provided either for free or at a cost.

In addition, we also found that the impact on students’ acceptance of vision care and eyeglasses varied among the three different incentive subgroups. High-value teacher incentives and moderate-value teacher incentives both had a significant impact on their students’ acceptance of offered vision care; however, cash incentives for teachers did not affect their students’ acceptance of offered vision care. These results may have been impacted by the fact that teachers were notified about the monetary value of each incentive. It may also be because material incentives (in this study, the sunglasses and the eyeglasses) are considered by teachers as gifts rather than as commissions earned when a corresponding task is completed. Due to the particular respect towards and societal expectations placed on teachers in China, teachers tend to avoid actively completing tasks that include cash incentives. These results suggest that material incentives rather than monetary ones might be better incentives to give teachers in rural China.

We believe that the results of the present study have important implications for future vision care programs for school-aged children in rural areas. Our findings suggest that, based on a vision healthcare model implemented in one of China’s poor rural counties, teacher incentives significantly improve the acceptance of offered vision care by their students with uncorrected refractive error.

This study has three strengths. First is its randomized controlled design and randomly selected cohort from a social group at risk for myopia. Second, we included both primary and junior high school students, as we believe it important to examine the gap between these two critical age groups due to myopia increasing in prevalence with age [34]. As our results showed that providing incentives to teachers has a significant impact on student acceptance of offered vision care for both schooling levels, we believe our results are robust. Third, this study’s successful collaboration with the local county hospital and Bureaus of Education to establish the VC demonstrates its relevance for future vision care policies and government initiatives.

This study also has some limitations. First, all our sample schools were from one county in rural China, limiting the study’s external validity. Second, due to limited time during the screening process, the vision screening of students was conducted by both professional VC staff and by local teachers. It is for this reason that we cannot separate individual impacts between these two groups. Future studies are needed to investigate the potentially distinct impacts of a screening conducted entirely by local teachers. Third, the intervention group had more primary school students with a higher acceptance of offered vision care, which may overestimate actual behavior. We note, however, that the observed impact is still present when we examined the OR for primary schools and junior high schools separately. Additionally, the impact of the intervention may be underestimated, as parents and students could elect to purchase eyeglasses at local optical shops or elsewhere on their own.

Last but not least, this study, for practical reasons, had to be carried out with a randomized cluster design rather than randomizing the teachers or students, and this scheme has some inherent limitations. In this case, it seems to have led to some differences between the control and intervention groups. For example, there are some potential characteristics (e.g., student age and their parent’s migration status) that were not fully balanced by randomization. For these reasons, our results must be applied to other settings with caution.

## 5. Conclusions

This study makes a valuable contribution to the literature by testing the effect of teacher incentives on students with uncorrected refractive errors’ acceptance of offered vision care. Teacher incentives can raise the uptake of underutilized health services in China, such as vision care, as well as complement existing findings that suggest awareness of a possible health risk alone is insufficient to convince vulnerable groups to seek out healthcare [33]. This study’s findings confirm the significant value of incentive mechanisms when seeking to raise the uptake of underutilized health services. Incentives may be effective in encouraging the uptake of vision healthcare for students in China’s rural areas, as well as reduce the burden of uncorrected refractive error among students of other under-developed areas with a high prevalence of refractive error.

## Figures and Tables

**Table 1:** Baseline characteristics of students with uncorrected refractive errors, by group assignment.

	Full Sample (n = 3401)			Analytic Sample (n = 3224)			
Variable	Control	Intervention	*p*-Value	Control	Intervention	*p*-Value	Missing Data, n
	n = 1645	n = 1765		n = 1579	n = 1645		
**Student characteristics**							
Age, mean (SD), year	12.1 (1.5)	11.7 (1.9)	<0.001	12.1 (1.5)	11.8 (1.9)	<0.001	0 (0.0)
Female sex, n (%)	854 (51.9)	950 (54.1)	0.202	818 (51.8)	897 (54.5)	0.121	0 (0.0)
Eyeglasses owned at baseline, n (%)	636 (38.7)	631 (35.9)	0.100	610 (38.6)	598 (36.4)	0.182	0 (0.0)
Visual acuity at baseline, mean (SD), LogMAR (Snellen fraction)	0.48 (0.2)	0.51 (0.2)	<0.001	0.48 (0.2)	0.51 (0.2)	<0.001	0 (0.0)
At least 1 parent with ≥9 years education, n (%)	1365 (83.0)	1378 (78.5)	0.045	1344 (85.1)	1357 (82.5)	0.053	127 (3.7)
At least 1 parent out-migrated for work, n (%)	885 (53.8)	1047 (59.6)	<0.001	872 (55.2)	1012 (61.5)	<0.001	117 (3.4)
Attending primary school (versus junior high), n (%)	471 (28.6)	971 (55.3)	<0.001	445 (28.2)	879 (53.4)	<0.001	0 (0.0)
**Town characteristics**							
Distance between town and county seat, mean (SD), km	14.13 (14.9)	18.07 (13.1)	<0.001	14.12 (14.8}	18.03 (13.0)	<0.001	0 (0.0)
**Teacher characteristics**							
Teaching experience, mean (SD), year	14.29 (6.2)	14.39 (7.5)	0.723	14.32 (6.2)	14.33 (7.5)	0.964	0 (0.0)
Teacher supports myopic students wearing eyeglasses, n (%)	1349 (82.0)	1337 (76.1)	0.371	1298 (82.2)	1250 (76.0)	0.343	0 (0.0)
Teacher believes that eye exercises cannot prevent myopia, n (%)	483 (29.4)	481 (27.4)	0.203	461 (29.2)	435 (26.4)	0.081	0 (0.0)
Teacher believes that myopic students not wearing eyeglasses worsens school performance, n (%)	1476 (89.7)	1585 (90.3)	0.603	1411 (89.4)	1480 (89.0)	0.570	0 (0.0)
Teacher believes that students with low degrees of myopia should wear eyeglasses, n (%)	904 (55.0)	902 (52.34)	0.134	861 (54.5)	858 (52.2)	0.178	0 (0.0)

**Table 2:** Descriptive statistical analysis of the impact of providing teacher incentives on students’ acceptance of offered vision care and on the eyeglasses uptake rate.

**Impact of Providing Teacher Incentives on Students’ Acceptance of Offered Vision Care**				
**Group**	**Full Sample, n (%)**	**Acceptance of Offered Vision Care, n (%)**	**Mean Difference Compared to Control Group**	***p*-Value**
Control group	1579 (49.0)	172/1579 (10.9)		
Intervention group	1645 (51.0)	382/1645 (23.2)	12.3	<0.001
High-value incentive (sunglasses worth USD 148)	524 (31.9)	144/524 (27.5)	16.6	<0.001
Moderate-value incentive (eyeglasses worth USD 89)	582 (35.4)	149/539 (27.6)	16.7	<0.001
Cash incentive (USD 35)	539 (32.8)	89/582 (15.3)	4.4	0.182
**Eyeglasses Uptake Rate of Students Who Accepted Offered Vision Care**				
**Group**	**The Children Who Needed Eyeglasses, n (%)**	**Eyeglasses Uptake, n (%)**	**Mean Difference Compared to Control Group**	***p*-Value**
No teacher incentive	161/172 (93.6)	135/161 (85.6)		
Teacher incentive	347/382 (90.8)	268/347 (77.2)	−8.4	0.042
High-value incentive (sunglasses worth USD 148)	131/144 (91.0)	105/131 (80.2)	−5.4	0.957
Moderate-value incentive (eyeglasses worth USD 89)	133/149 (89.3)	110/133 (82.7)	−2.9	0.729
Cash incentive (USD 35)	83/89 (93.3)	53/83 (63.9)	−21.7	0.002

**Table 3:** Logistic regression analysis of the impact of providing teacher incentives on students’ acceptance of offered vision care.

	Acceptance of Offered Vision Care								
Variable	Univariate Analysis n = 3224			Multivariate Analysis n = 3224			Multivariate Analysis n = 3224		
	OR ^a^	95% CI ^b^	*p*-Value	OR ^a^	95% CI ^b^	*p*-Value	OR ^a^	95% CI ^b^	*p*-Value
Intervention group	2.47	2.03–3.01	<0.001	1.94	1.57–2.38	<0.001	2.46	2.02–2.99	<0.001
Student characteristics									
Age, year	0.73	0.69–0.77	<0.001	0.89	0.81–0.99	0.028			
Female sex	1.35	1.12–1.63	0.001	1.36	1.12–1.65	0.002	1.35	1.12–1.63	0.002
Eyeglasses owned at baseline	0.74	0.61–0.89	0.002	1.00	0.81–1.24	0.978	0.74	0.61–0.90	0.004
Visual acuity at baseline (LogMAR)	2.31	1.52–3.31	<0.001	1.95	1.25–3.04	0.003			
At least 1 parent with ≥9 years education	0.87	0.68–1.10	0.248	0.94	0.73–1.21	0.648	0.88	0.68–1.13	0.328
At least 1 parent out-migrated for work	0.91	0.75–1.09	0.309	0.81	0.67–0.99	0.038			
Attending primary school (versus junior high)	3.34	2.76–4.51	<0.001	2.04	1.40–2.98	<0.001			
Town characteristics									
Distance between town and county seat, km	1.01	1.01–1.02	<0.001	1.01	1.00–1.02	0.016			
Teacher characteristics									
Teaching experience, year	1.02	1.00–1.03	0.013	1.00	0.99–1.02	0.674	1.01	0.99–1.03	0.085
Teacher supports myopic students wearing eyeglasses	1.18	0.94–1.49	0.164	1.16	0.89–1.50	0.267	1.25	0.97–1.62	0.088
Teacher believes that eye exercises cannot prevent myopia	1.03	0.84–1.27	0.752	1.09	0.87–1.36	0.452	1.01	0.81–1.26	0.921
Teacher believes that myopic students not wearing eyeglasses worsens school performance	1.80	1.26–2.58	0.001	1.49	1.02–2.18	0.037	1.66	1.16–2.39	0.006
Teacher believes that students with low degrees of myopia should wear eyeglasses	0.95	0.79–1.15	0.614	0.88	0.71–1.08	0.217	0.92	0.75–1.13	0.426

Note: Data source: baseline survey. ^a^ OR = odds ratio; ^b^ CI = 95% confidence interval, reported in parentheses.

**Table 4:** Logistic regression analysis of the impact of providing teacher incentives on students’ acceptance of offered vision care between primary and junior high school groups.

	Acceptance of Offered Vision Care											
	Primary Schools						Junior High Schools					
Variable	Univariate Analysis n = 1324			Multivariate Analysis n = 1324			Univariate Analysis n = 1900			Multivariate Analysis n = 1900		
	OR ^a^	95% CI ^b^	*p*-Value	OR ^a^	95% CI ^b^	*p*-Value	OR ^a^	95% CI ^b^	*p*-Value	OR ^a^	95% CI ^b^	*p*-Value
Intervention group	2.19	1.66–2.91	<0.001	2.18	1.64–2.90	<0.001	1.66	1.23–2.24	0.001	1.89	1.35–2.66	<0.001
**Student characteristics**												
Age, mean (SD), year				1.07	0.93–1.25	0.345				0.75	0.64–0.88	<0.001
Female sex				1.35	1.05–1.73	0.020				1.36	0.99–1.86	0.058
Eyeglasses ownership at baseline				0.90	0.67–1.21	0.479				1.05	0.77–1.44	0.758
Visual acuity at baseline (LogMAR)				2.22	1.22–4.04	0.009				1.63	0.82–3.26	0.162
At least 1 parent with ≥9 years education				0.95	0.69–1.31	0.776				0.98	0.64–1.49	0.916
At least 1 parent out-migrated for work				0.82	0.64–1.06	0.138				0.80	0.58–1.09	0.151
**Town characteristics**												
Distance between the town and county seat, km				1.01	1.00–1.02	0.171				1.02	1.00–1.03	0.012
**Teacher characteristics**												
Teaching experience, years				1.01	0.99–1.02	0.553				1.01	0.98–1.03	0.690
Teacher supports myopic students wearing eyeglasses				0.91	0.65–1.28	0.593				1.82	1.19–2.79	0.006
Teacher believes that eye exercises cannot prevent myopia				1.25	0.93–1.69	0.132				0.70	0.49–1.02	0.063
Teacher believes that myopic students not wearing eyeglasses worsens school performance				1.37	0.81–2.31	0.245				1.62	0.91–2.87	0.099
Teacher believes that students with low degrees of myopia should wear eyeglasses				0.95	0.72–1.26	0.722				0.81	0.58–1.14	0.225

Note: Data source: baseline survey. ^a^ OR = odds ratio; ^b^ CI = 95% confidence interval, reported in parentheses.

**Table 5:** Logistic regression analysis of the impact of providing different teacher incentives on students’ acceptance of offered vision care.

	Acceptance of Offered Vision Care											
Variable	Univariate Analysis n = 3224			Multivariate Analysis n = 3224			Multivariate Analysis n = 3224			Multivariate Analysis n = 3224		
	OR ^a^	95% CI ^b^	*p*-Value	OR ^a^	95% CI ^b^	*p*-value	OR ^a^	95% CI ^b^	*p*-Value	OR ^a^	95% CI ^b^	*p*-Value
High-value incentive	2.12	1.70–2.63	<0.001	1.67	1.32–2.11	< 0.001						
Moderate-value incentive	2.15	1.73–2.67	<0.001				1.78	1.42–2.23	<0.001			
Cash incentive	0.85	0.66–1.08	0.182							0.80	0.61–1.05	0.114
**Student characteristics**												
Age, mean (SD), year				0.92	0.83–1.02	0.097	0.93	0.84–1.03	0.157	0.94	0.85–1.03	0.187
Female sex				1.35	1.11–1.64	0.002	1.38	1.14–1.68	0.001	1.37	1.13–1.67	0.001
Eyeglasses owned at baseline				1.01	0.82–1.25	0.901	1.02	0.82–1.26	0.868	1.02	0.83–1.26	0.835
Visual acuity at baseline (LogMAR)				2.08	1.34–3.24	0.001	2.12	1.37–3.30	0.001	2.18	1.40–3.39	0.001
At least 1 parent with ≥9 years education				0.95	0.74–1.22	0.666	0.96	0.75–1.24	0.762	0.95	0.74–1.23	0.703
At least 1 parent out-migrated for work				0.82	0.67–0.99	0.043	0.83	0.68–1.00	0.055	0.83	0.69–1.01	0.068
Attending primary school				2.46	1.72–3.53	<0.001	2.52	1.75–3.63	<0.001	2.77	1.94–3.97	<0.001
**Town characteristics**												
Distance between town and county seat, km				1.01	1.00–1.01	0.020	1.01	1.00–1.02	0.004	1.01	1.00–1.02	0.009
**Teacher characteristics**												
Teaching experience, years				1.00	0.99–1.02	0.591	1.00	0.99–1.02	0.794	1.00	0.99–1.02	0.743
Teacher supports myopic students wearing eyeglasses				1.19	0.92–1.54	0.194	1.18	0.92–1.51	0.202	1.16	0.90–1.49	0.253
Teacher believes that eye exercises cannot prevent myopia				1.13	0.91–1.41	0.266	1.11	0.89–1.38	0.365	1.11	0.89–1.39	0.338
Teacher believes that myopic students not wearing eyeglasses worsens school performance				1.49	1.02–2.17	0.039	1.41	0.97–2.06	0.075	1.45	1.00–2.12	0.052
Teacher believes that students with low degrees of myopia should wear eyeglasses				0.85	0.68–1.05	0.121	0.88	0.72–1.09	0.235	0.85	0.69–1.05	0.132

Note: Data source: baseline survey. ^a^ OR = odds ratio; ^b^ CI = 95% confidence interval, reported in parentheses.

## Data Availability

The data that support the findings of this study are available from the corresponding author upon reasonable request.

## References

[1] Listed N. (2006). Sight Test and Glasses Could Dramatically Improve the Lives of 150 Million People with Poor Vision. Indian J. Med. Sci..

[2] He M., Zeng J., Liu Y., Xu J., Pokharel G.P., Ellwein L.B. (2004). Refractive Error and Visual Impairment in Urban Children in Southern China. Investig. Opthalmol. Vis. Sci..

[3] He M., Huang W., Zheng Y., Huang L., Ellwein L.B. (2007). Refractive error and visual impairment in school children in rural southern China. Ophthalmology.

[4] Yi H., Zhang L., Ma X., Congdon N., Shi Y., Pang X., Zeng J., Wang L., Boswell M., Rozelle S. (2015). Poor vision among China’s rural primary school students: Prevalence, correlates and consequences. China Econ. Rev..

[5] Resnikoff S. (2008). Global magnitude of visual impairment caused by uncorrected refractive errors in 2004. Bull. World Health Organ..

[6] Ma X., Zhou Z., Yi H., Pang X., Shi Y., Chen Q., Meltzer M.E., Le Cessie S., He M., Rozelle S. (2014). Effect of providing free glasses on children’s educational outcomes in China: Cluster randomized controlled trial. BMJ.

[7] Glewwe P., Park A., Zhao M. (2016). A better vision for development: Eyeglasses and academic performance in rural primary schools in China. J. Dev. Econ..

[8] Congdon N., Wang Y., Song Y., Choi K., Zhang M., Zhou Z., Xie Z., Li L., Liu X., Sharma A. (2008). Visual Disability, Visual Function, and Myopia among Rural Chinese Secondary School Children: The Xichang Pediatric Refractive Error Study (X-PRES)—Report 1. Investig. Opthalmol. Vis. Sci..

[9] Krishnaratne S., White H., Carpenter E., International Initiative for Impact Evaluation (3ie) Quality Education for All Children? What Works in Education in Developing Countries.

[10] Wang X., Congdon N., Ma Y., Hu M., Zhou Y., Liao W., Jin L., Xiao B., Wu X., Ni M. (2017). Cluster-randomized controlled trial of the effects of free glasses on purchase of children’s glasses in China: The PRICE (Potentiating Rural Investment in Children’s Eyecare) study. PLoS ONE.

[11] Wang X., Yi H., Lu L., Zhang L., Ma X., Jin L., Zhang H., Naidoo K.S., Minto H., Zou H. (2015). Population Prevalence of Need for Spectacles and Spectacle Ownership Among Urban Migrant Children in Eastern China. JAMA Ophthalmol..

[12] Congdon N., Li L., Zhang M., Yang A., Gao Y., Griffiths S., Wu J., Sharma A., Lam D.S.C. (2011). Randomized, Controlled Trial of an Educational Intervention to Promote Spectacle Use in Rural China. Ophthalmology.

[13] Bai Y., Yi H., Zhang L., Shi Y., Ma X., Congdon N., Zhou Z., Boswell M., Rozelle S. (2014). An investigation of vision problems and the vision care system in rural china. Southeast Asian J. Trop. Med. Public Health.

[14] Li L. (2010). Attitudes of Students, Parents, and Teachers Toward Glasses Use in Rural China. Arch. Ophthalmol..

[15] Li L., Song Y., Liu X., Lu B., Choi K., Lam D.S.C., Zhang M., Zheng M., Wang Y., Sharma A. (2008). Spectacle Acceptance among Secondary School Students in Rural China: The Xichang Pediatric Refractive Error Study (X-PRES)—Report 5. Investig. Opthalmol. Vis. Sci..

[16] Zhou Z., Zeng J., Ma X., Pang X., Yi H., Chen Q., Meltzer M.E., He M., Rozelle S., Congdon N. (2014). Accuracy of Rural Refractionists in Western China. Investig. Opthalmol. Vis. Sci..

[17] Guan H., Wang H., Huang J., Du K., Zhao J., Boswell M., Shi Y., Iyer M., Rozelle S. (2018). Health Seeking Behavior among Rural Left-Behind Children: Evidence from Shaanxi and Gansu Provinces in China. Int. J. Environ. Res. Public Health.

[18] CAPABILITIES. https://www.brienholdenvision.org/translational-research/capabilities.html.

[19] Vision Centre Model—IAPB. https://www.iapb.org/resources/vision-centre-model/.

[20] Jan C.L., Congdon N. (2018). Chinese national policy initiative for the management of childhood myopia. Lancet. Child Adolesc. Health.

[21] Ma Y., Gao Y., Wang Y., Li H., Ma L., Jing J., Shi Y., Guan H., Congdon N. (2018). Impact of a Local Vision Care Center on Glasses Ownership and Wearing Behavior in Northwestern Rural China: A Cluster-Randomized Controlled Trial. Int. J. Environ. Res. Public Health.

[22] Help End Avoidable Blindness // Fred Hollows Foundation. https://www.hollows.org/au/what-we-do/ending-avoidable-blindness.

[23] Ma Y., Congdon N., Shi Y., Hogg R., Medina A., Boswell M., Rozelle S., Iyer M. (2018). Effect of a Local Vision Care Center on Eyeglasses Use and School Performance in Rural China: A Cluster Randomized Clinical Trial. JAMA Ophthalmol..

[24] Adams K.S., Christenson S.L. (2000). Trust and the Family–School Relationship Examination of Parent–Teacher Differences in Elementary and Secondary Grades. J. Sch. Psychol..

[25] Cascella P.W., Trief E., Bruce S.M. (2012). Parent and Teacher Ratings of Communication Among Children With Severe Disabilities and Visual Impairment/Blindness. Commun. Disord. Q..

[26] Wang X., Ma Y., Hu M., Jin L., Xiao B., Ni M., Congdon N. (2018). Varga Beatrice Teachers’ influence on purchase and wear of children’s glasses in rural China: The PRICE study. Clin. Exp. Ophthalmol..

[27] Yi H., Zhang H., Ma X., Zhang L., Wang X., Jin L., Naidoo K., Minto H., Zou H., Lu L. (2015). Impact of Free Glasses and a Teacher Incentive on Children’s Use of Eyeglasses: A Cluster-Randomized Controlled Trial. Am. J. Ophthalmol..

[28] Du K., Huang J., Guan H., Zhao J., Zhang Y., Shi Y. (2022). Teacher-to-parent communication and vision care-seeking behaviour among primary school students. Hong Kong Med. J..

[29] Sharma A., Congdon N., Patel M., Gilbert C. (2012). School-based Approaches to the Correction of Refractive Error in Children. Surv. Ophthalmol..

[30] Sharma A. (2008). Strategies to Improve the Accuracy of Vision Measurement by Teachers in Rural Chinese Secondary Schoolchildren: Xichang Pediatric Refractive Error Study (X-PRES) Report No. 6. Arch. Ophthalmol..

[31] Shaanxi Statistical Yearbook 2015. http://www.shaanxitj.gov.cn/upload/2016/tongjinianj/2015/indexch.htm.

[32] National Bureau of Statistics: China Statistical Yearbook 2015. http://data.stats.gov.cn/easyquery.htm?cn=E0103.

[33] Chadha R.K., Subramanian A. (2011). The effect of visual impairment on quality of life of children aged 3-16 years. Br. J. Ophthalmol..

[34] Miguel E., Kremer M. (2004). Worms: Identifying Impacts on Education and Health in the Presence of Treatment Externalities. Econometrica.

